# MicroRNA cluster 7/17/155 as a potential driver for high-grade breast tumors transformation through EGFR-associated EMT

**DOI:** 10.3389/fcell.2026.1899163

**Published:** 2026-08-14

**Authors:** Saeed Khodayari, Hamid Khodayari, Hanieh Jalali, Elnaz Saeedi, Ahad Muhammadnejad, Mohammad Dashtkoohi, Amirnader Emami Razavi, Meghdad Yeganeh, Reza Shirkoohi, Seyed Rouhollah Miri, Ramesh Omranipour, Habibollah Mahmoodzadeh

**Affiliations:** 1 Breast Disease Research Center, Cancer Institute of Iran, Imam Khomeini Hospital Complex, Tehran University of Medical Sciences, Tehran, Iran; 2 Department of Animal Biology, Faculty of Biological Sciences, Kharazmi University, Tehran, Iran; 3 Oxford Clinical Trials Research Unit, Nuffield Department of Orthopaedics, Rheumatology and Musculoskeletal Sciences, University of Oxford, Oxford, United Kingdom; 4 Cancer Biology Research Center, Cancer Institute of Iran, Tehran University of Medical Sciences, Tehran, Iran; 5 Iran National Tumor Bank, Cancer Institute of Iran, Imam Khomeini Hospital Complex, Tehran University of Medical Sciences, Tehran, Iran; 6 School of Biotechnology, College of Science, University of Tehran, Tehran, Iran; 7 Department of Surgical Oncology, Cancer Institute, Imam Khomeini Hospital Complex, Tehran University of Medical Science, Tehran, Iran

**Keywords:** breast cancer, EGFR, EMT, high-grade tumor, MicroRNAs 7/17/155, single-cell RNA sequencing

## Abstract

**Background:**

Breast tumor dedifferentiation and progression onto high-grade states with stem-like fea-tures are governed by complex regulatory networks within the tumor microenvironment. This study aimed to identify a potential microRNA (miRNA) cluster associated with breast tumor stemness and in-vestigate its regulatory functions inspired by mammary gland development (MGD).

**Methods:**

We reana-lyzed microarray data and identified differentially expressed mRNAs and miRNAs in breast cancer of various grades. High-throughput transcriptomic datasets were incorporated to strengthen the discovery framework. Spatial mRNA–miRNA interactions in each breast cancer grade were identified, and a series of q-PCR, immunohistochemical analysis of epidermal growth factor receptor (EGFR) expression, and Single-cell RNA sequencing were used to validate findings.

**Results:**

MGD-related regulatory mecha-nisms correlated with well- and moderately-differentiated tumors but were absent in high-grade tumors. miRNAs 7, 17, and 155 were significantly upregulated in undifferentiated high-grade tumors. These tu-mors also showed marked activation of EGFR-mediated epithelial-mesenchymal transition (EMT) and elevated histological EGFR levels. scRNA-seq revealed a stem cell-like and undifferentiated cellular pop-ulation characterized by miRNAs 7, 17, and 155, as well as EGFR-associated EMT activation. Pseudotemporal trajectory analysis indicated this cluster as a potential origin of malignant breast tumor heterogeneity and stemness.

**Conclusion:**

The miRNA cluster 7/17/155 may act as a potential driver of breast tumor dedifferentiation and acquisition of stem-like properties via EGFR-mediated EMT. This cluster represents a promising target for therapies to reverse tumor dedifferentiation and a biomarker for molecular classification of breast tumor differentiation status.

## 1 Introduction

Mammary glands (MGs) are specialized organs responsible for milk production during lactation, which consist of ductal and lobular structures lined by luminal epithelium (Slepicka et al., 2021; Dawson and Visvader, 2021). MGs development in females occurs in four distinct stages: embryonic, puberty, pregnancy–lactation, and involution (Dawson and Visvader, 2021; Slepicka et al., 2021; Watson and Khaled, 2008). Transitions between these phases are driven by dynamic changes in paracrine and growth factor signaling (Watson and Khaled, 2008; Slepicka et al., 2021). Following the prepubertal development stage, estrogen and epidermal growth factor (EGF) stimulate neoductogenesis and terminal bud formation. During maturation, ductal morphogenesis is regulated by progesterone and insulin-like growth factors 1 and 2 (IGF-1/2), in which extensive lateral branching is prompted (Fu et al., 2020). Prolactin production during pregnancy and oxytocin during lactation activate pathways, such as GATA binding protein 3 (GATA3), signal transducer and activator of transcription 5A (STAT5A), and CCAAT/enhancer-binding protein β (C/EBPβ), which encourage alveologenesis and lactation differentiation (Watson and Khaled, 2008; Khodayari et al., 2023). Finally, IGF binding protein 5 (IGFBP5) and transforming growth factor β3 (TGF-β3) mediate alveolar regression during an involution-like process (Slepicka et al., 2021; Fu et al., 2020). Recent studies have supported the hypothesis that molecular processes regulating cell development and differentiation of MGs may be linked to the onset of breast carcinomas.

A carcinoma refers to a malignant epithelial tumor and accounts for the majority of MG malignancies (Farhanji et al., 2015). Breast carcinoma cells exhibit abnormal phenotypic characteristics that correspond to their level of similarity to the original cell type, a concept referred to as differentiation status. Based on these features, tumors are histologically categorized into three grades: Grade I (GI) tumors are well-differentiated, Grade II (GII) tumors are moderately differentiated, and Grade III (GIII) tumors display stemness phenotypes with undifferentiated morphology (Wang et al., 2020). Breast cancer remains a formidable challenge in oncology, largely due to the clinical aggressiveness of high-grade, undifferentiated tumors. These Grade III (GIII) malignancies are characterized by stem-like phenotypes, enhanced invasiveness, and resistance to therapy, which collectively drive poor patient outcomes (Wang et al., 2020; Adorno-Cruz et al., 2021). A pivotal, yet incompletely understood, process in their pathogenesis is tumor cell dedifferentiation—the regression to a less specialized, stem-cell-like state (Barua et al., 2020).

A key mechanism enabling cellular plasticity in both development and cancer is the epithelial-mesenchymal transition (EMT) (Khodayari et al., 2025). EMT can be induced by various signals, with the epidermal growth factor receptor (EGFR) pathway being a potent trigger (Wendt et al., 2010; Ali and Wendt, 2017). Activation of the EGF receptor (EGFR) in breast carcinoma cells has been shown to trigger downstream signaling pathways, including mitogen-activated protein kinase (MAPK), Ak strain transforming (AKT), and signal transducer and activator of transcription (STAT), thereby facilitating EMT induction in epithelial cells (Ali and Wendt, 2017). However, the EGFR-EMT axis alone cannot fully explain the complex reprogramming of breast cancer cells (Pasquier et al., 2015), pointing to the involvement of additional regulatory layers.

MicroRNAs (miRNAs) represent a critical layer of post-transcriptional control and are established regulators of both normal MGD and breast cancer progression (Isanejad et al., 2016; Khori et al., 2015; Wang et al., 2021a; Jiang et al., 2022). Notably, miRNAs involved in mammary differentiation, such as those in the miR-212/132 family, are often downregulated in high-grade tumors (Damavandi et al., 2016). These findings suggest that miRNAs implicated in MGD may also play a role in regulating the emergence of undifferentiated phenotypes in breast cancer. Therefore, this study aims to bridge developmental biology and cancer pathogenesis by leveraging MGD signatures to identify key miRNA regulators of breast tumor grading. To achieve this, we integrate high-throughput transcriptomic analyses (microarray and single-cell RNA sequencing) with experimental validation to test the central hypothesis that a specific miRNA cluster—miR-7/17/155—functions as a driver of breast tumor dedifferentiation by augmenting EGFR-mediated EMT, thereby promoting the emergence of aggressive, high-grade disease.

## 2 Materials and methods

### 2.1 Microarray datasets collection and differentially expressed mRNAs/miRNAs analysis

mRNA expression data were obtained from the Gene Expression Omnibus (GEO, http://www.ncbi.nlm.nih.gov/geo) database, specifically from the GSE29044 dataset. Microarray analysis was performed using the Affymetrix Human Genome U133 Plus 2.0 platform (GPL570). A total of 92 breast cancer samples, including 32 normal, 3 GI, 36 GII, and 26 GIII, were collected for mRNA expression analysis. The miRNA expression data were obtained from the ArrayExpress (https://www.ebi.ac.uk/arrayexpress/experiments/browse.html) database, specifically from the GSE45666 dataset. 75 breast cancer samples, including 11 normal, 8 GI, 26 GII, and 30 GIII, were collected for differential miRNA expression analysis.

Differential expression of genes (DEGs) and miRNAs (DEMs) was analyzed using the GEO2R online software. The DEGs and DEMs between all three grades of breast carcinoma and normal tissue were identified based on a P value ≤0.05 and by considering the upregulated (positive LogFc) and downregulated (negative LogFc) miRNAs and mRNAs. Significant DEMs and DEGs were visualized using Volcano plots created within the VolcaNoseR software (https://goedhart.shinyapps.io/VolcaNoseR). The FunRich software (version 3.1.3) was used for a Venn analysis to detect significant DEMS and DEGs.

### 2.2 Pathway and gene ontology enrichment analysis

The EnrichR tool (https://maayanlab.cloud/Enrichr) was used to analyze the enrichment of KEGG pathways, WIKI pathways, and Gene Ontology (GO) of the upregulated and downregulated DEGs. The five most significant pathways in each category were visualized using -Log10 (P-value) and odds ratio. To enrich KEGG pathways for the upregulated and downregulated DEMs, the DIANA-miRPath v3 tool (https://dianalab.e-ce.uth.gr/html/mirpathv3/index.php?r=mirpath) was used (Denaro et al., 2019). The five most active pathways of each category were visualized using -Log10 (P-value). The pathway enrichment analysis of target miRNAs was performed using the MiRWALK software (http://mirwalk.umm.uni-heidelberg.de/search_mirnas). The enrichment results for KEGG pathways and biological processes were visualized using SRplot (https://www.bioinformatics.com.cn).

### 2.3 MGD involved mRNA detection

The results of WIKI pathway and biological process enrichment in significantly upregulated and downregulated genes (P ≤ 0.05) were used to identify DEGs of mammary gland development in different stages and their target mRNAs in GI, GII, and GIII breast tumors. The included MGD stages in WIKI pathway enrichment were embryonic development (WP2813), puberty (WP2814), pregnancy and lactation (WP2817), and involution (WP2815). The other mechanisms of interest in GO biological process enrichment were mammary gland development (GO:0030879), mammary gland epithelial development (GO:0061180), mammary gland epithelial cell differentiation (GO:0060644), and regulation of mammary gland epithelial cell proliferation (GO:0033599). Sankey diagrams of the discovered active and suppressed MGD mechanisms in GI, GII, and GIII breast tumors were generated using the SankeyMATIC online tool (https://sankeymatic.com/). Heat maps were also generated to visualize target mRNA expression using SRplot, based on the results of GSE29044 gene expression analysis for the three different grades.

### 2.4 miRNA-mRNA integrated analysis

The breast carcinoma platform of miRNet 2.0 (https://www.mirnet.ca/miRNet/home.xhtml) was used to detect and visualize the miRNA-mRNA interaction network between the upregulated mRNAs of each breast carcinoma grade’s target upregulated mRNAs with significantly (P ≤ 0.05) upregulated DEMs.

### 2.5 Target miRNAs expression validation and correlation analysis

We utilized the samples of the GSE45666 dataset (with the normalized expression value of Log2) to determine the relatively downregulated miRNAs. For this analysis, we collected a total of 111 tissue samples, including 15 normal samples, 10 GI, 41 GII, and 45 GIII breast tumor samples. The relative expression analysis was conducted using miRNA-16-5p as the reference miRNA (Rinnerthaler et al., 2016). We utilized RStudio software with ggplot2 and Circlize packages to generate violin and circular plots of the miRNA expression values. After selecting miRNA candidates, the radar chart of their expression was visualized using the Flourish Studio app (https://app.flourish.studio/@flourish/radar).

### 2.6 Ethical considerations and sample preparation

The experimental phase of this study involved a cohort of 27 female patients who had recently been diagnosed with breast carcinoma. The study was conducted between February and March 2022 at the Cancer Institute of Iran, affiliated with Tehran University of Medical Sciences (Tehran, Iran). Tumor and normal breast tissue samples were collected through the Iran National Tumor Bank at the Cancer Institute of Iran during this period. All participants were over the age of 20 and provided written informed consent before undergoing ultrasound-guided biopsies using a 14-gauge core needle. A total of 6 GI, 11 GII, and 10 GIII tumor samples, along with 15 normal breast tissues, were obtained and immediately stored in liquid nitrogen for downstream molecular analyses. Tumor grading was performed by a certified pathologist using hematoxylin and eosin (H&E) staining in accordance with the Nottingham grading system. IHC was applied for tumor subtyping. All procedures were conducted in compliance with institutional and national ethical guidelines and were approved by the Institutional Review Board of Tehran University of Medical Sciences (ethical code: IR. TUMS.IKHC.REC.1400.506). The demographic and clinicopathological characteristics of the patients are summarized in Supplementary Table S1.

### 2.7 Quantitative real-time PCR (qRT-PCR)

Total RNA was extracted from tissue samples using the FavorPrep™ miRNA Extraction Kit (Favorgen, Taiwan) according to the manufacturer’s instructions. The quality and quantity of the isolated RNA were assessed by spectrophotometric analysis and agarose gel electrophoresis. The RNA samples were stored at −80 °C until further use. For cDNA synthesis, 10 µL of the total RNA was reverse-transcribed in a 20 µL reaction volume using the BONmiR 1st-strand cDNA synthesis kit (Bonyakhteh, Tehran, Iran) following the manufacturer’s recommendations. Quantification of the target miRNAs was performed by Real-Time PCR using SYBR Green qPCR Master Mix (Invitrogen Life Technologies) on the ABI Prism 7900HT system (Applied Biosystems, Massachusetts, USA) using the specific primers. Each reaction was carried out in triplicate and the primer sequences are provided in Supplementary Table S2. The levels of miRs were normalized using miR-16 as the endogenous control using the 2^−ΔΔCT^ method (Niu et al., 2016). ΔCT was calculated by subtracting the CT values of miR-16 from those of the target miRNAs, and ΔΔCT was determined by subtracting the mean ΔCT of the control group from that of the experimental group. Fold changes were calculated using equation 2^−ΔΔCT^.

The expression levels were compared with the healthy tissues and expressed as fold change, and the data visualization was performed in RStudio using the ggplot2 package.

### 2.8 Target miRNAs interaction and PPI network construction

The target mRNAs related to the identified miRNAs were isolated using the miRWalk database, which required a score of higher than 0.95. The interactions between these miRNAs and target mRNAs were further analyzed using the STRING database to identify common protein-protein interaction (PPI) networks. These PPI networks were then visualized using the Cytoscape software version 3.8.0, as shown in.

### 2.9 Gene set enrichment analysis of the EGFR-Mediated EMT on GI and GIII tumors

The Gene Set Enrichment Analysis (GSEA) software version 4.3.2 was utilized to conduct an EGFR-mediated EMT enrichment analysis. The analysis was performed using the log2 expression values of 24 GSE29044 data samples, which included 3 GI and 21 GIII samples. The EGFR-mediated EMT gene set was obtained from the study by Schinke et al. (Schinke et al., 2022). The PPI network was analyzed using the STRING database and visualized using the Cytoscape software version 3.8.0. Furthermore, the FunRich software version 3.1.3 was used to analyze the biological process enrichment of the EGFR-interacted mRNAs.

### 2.10 Survival analysis

The survival and prognostic value of microRNAs 7, 17, and 155 and EGFR expression levels for breast cancer patients were determined using the Kaplan-Meier Plotter database (http://kmplot.com/analysis). The significance of the differences was determined with a P-value less than 0.05.

### 2.11 Histopathology and IHC observation

A total of 16 formalin-fixed paraffin-embedded (FFPE) breast tissue samples, including 11 tumor specimens and 5 normal controls, were incorporated into a tissue microarray (TMA) using a previously published protocol (Zolfaghari et al., 2020). The margins of each TMA core were delineated based on H&E-stained sections prepared during routine diagnostic evaluation. Tumor grading was confirmed through histopathological examination of H&E-stained slides, following the standard pathological classification system for breast tumors (Rakha et al., 2023). According to this system, tumors are graded based on tubule formation, nuclear pleomorphism, and mitotic count, resulting in three categories:

- GI (well-differentiated): tumors with prominent tubule formation, mild nuclear atypia, and low mitotic activity.
- GII (moderately differentiated): tumors with intermediate features, including reduced tubule formation, moderate nuclear pleomorphism, and increased mitotic figures.
- GIII (poorly differentiated): tumors with minimal tubule formation, marked nuclear atypia, and high mitotic activity.

All FFPE samples were obtained from the Iran National Tumor Bank at the Cancer Institute of Tehran University of Medical Sciences. Information regarding the expression status of estrogen receptor (ER), progesterone receptor (PR), human epidermal growth factor receptor 2 (HER2), and Ki-67 was extracted from the corresponding medical records of each case. Detailed clinicopathological characteristics of the patients are summarized in Supplementary Table S3.

IHC staining of the TMA slides was performed according to standard protocols (Zolfaghari et al., 2020). Briefly, tissue sections (5 μm thick) were deparaffinized and dehydrated, followed by blocking of endogenous peroxidase activity using 3% hydrogen peroxide (v/v) for 20 min at room temperature. Antigen retrieval was carried out by autoclaving the sections three times at 121 °C in 10 mM sodium citrate buffer (pH 6.0). The slides were then incubated overnight with a primary monoclonal anti-EGFR/HER1 antibody (1:100 dilution; Abcam carcinomaam, UK). On the following day, the slides were treated with a secondary antibody cocktail (anti-rabbit/anti-mouse EnVision; Dako, Denmark) for 30 min. Detection was performed using 3,3-diaminobenzidine (DAB; Dako, Denmark), followed by counterstaining with hematoxylin (Dako, Denmark). The slides were subsequently dehydrated through graded alcohols, cleared in xylene (Dako, Denmark), and mounted for microscopic evaluation.

A blinded academic pathologist independently assessed immunostaining of EGFR. A semiquantitative scoring system was applied to determine the proportion of EGFR-positive tumor cells, categorized as follows: 0 = 0%, 1 = <1%, 2 = 1–10%, 3 = 11–33%, 4 = 34–66%, and 5 = >67%.

H&E and IHC-stained sections were examined under a light microscope (Olympus Provis, AX70, Japan), and representative images were captured using a digital camera (Olympus DP11, Japan) at magnifications of 4×, 10×, and 40×.

### 2.12 ScRNA-seq using seurat

ScRNAseq was performed on the breast carcinoma population using the count matrix of samples from the GSE180286 dataset. The standard cell clustering model was applied using the R package Seurat 4.3.0 (https://satijalab.org/seurat). Cells that had unique feature counts >5500 or <800, and >30% mitochondrial counts, were filtered out. After normalizing the data, a non-linear dimensional reduction of cells was performed using UMAP with default parameters. The DEGs in all clusters were obtained, and subsequently, a volcano plot of cluster 3 was generated using VolcaNoseR (https://huygens.science.uva.nl/VolcaNoseR2).

We obtained a list of target miRNAs related to the upregulated DEGs (averageLogfc >0.5) in cluster 3 from the miRWalk database. Then, we considered only those in the 3′UTR region with a binding score of >0.95 and extracted the results for hsa-miR-7, hsa-miR-17, and hsa-miR-155. Using EnrichR, we analyzed the biological processes related to the target mRNAs and visualized the crosstalk between miRNAs 7/17/155, cluster 3 DEGs, and the biological processes.

### 2.13 Single-cell trajectory analysis

The R package Monocle 3 (https://cole-trapnell-lab.github.io/monocle3) was used to order cells in a pseudo-time trajectory on our Seurat object. After reducing the dimensionality of the data and visualizing the results using the UMAP method, we ordered the cells based on their progression through the developmental program, as measured by Monocle3 in pseudo-time.

In addition, we performed trajectory inference analysis of cluster 3 tractions using the Asc-Seurat software (https://asc-seurat.readthedocs.io/en/latest/installation.html) to obtain the most prevalent genes through the cluster 3 cells projection. The Seurat object was first subsetted to contain only cluster 3 cells and then loaded into Asc-Seurat. We analyzed the trajectory process of the cluster 3 cells using a slingshot model.

### 2.14 Statistical analysis

Data were presented as mean ± standard deviation and analyzed using SPSS 26.0 software (IBM, USA). The ANOVA test was performed to compare the means between the groups, and the Tukey test was used as a *post hoc* test in our analysis. IHC expression of EGFR was performed using a nonparametric Kruskal–Wallis One-Way analysis between groups. We consider α = 0.05 as the lowest significant level for the analysis.

## 3 Results

### 3.1 Grade-specific DEGs/DEMs and their mediating mechanisms

**FIGURE 1:**
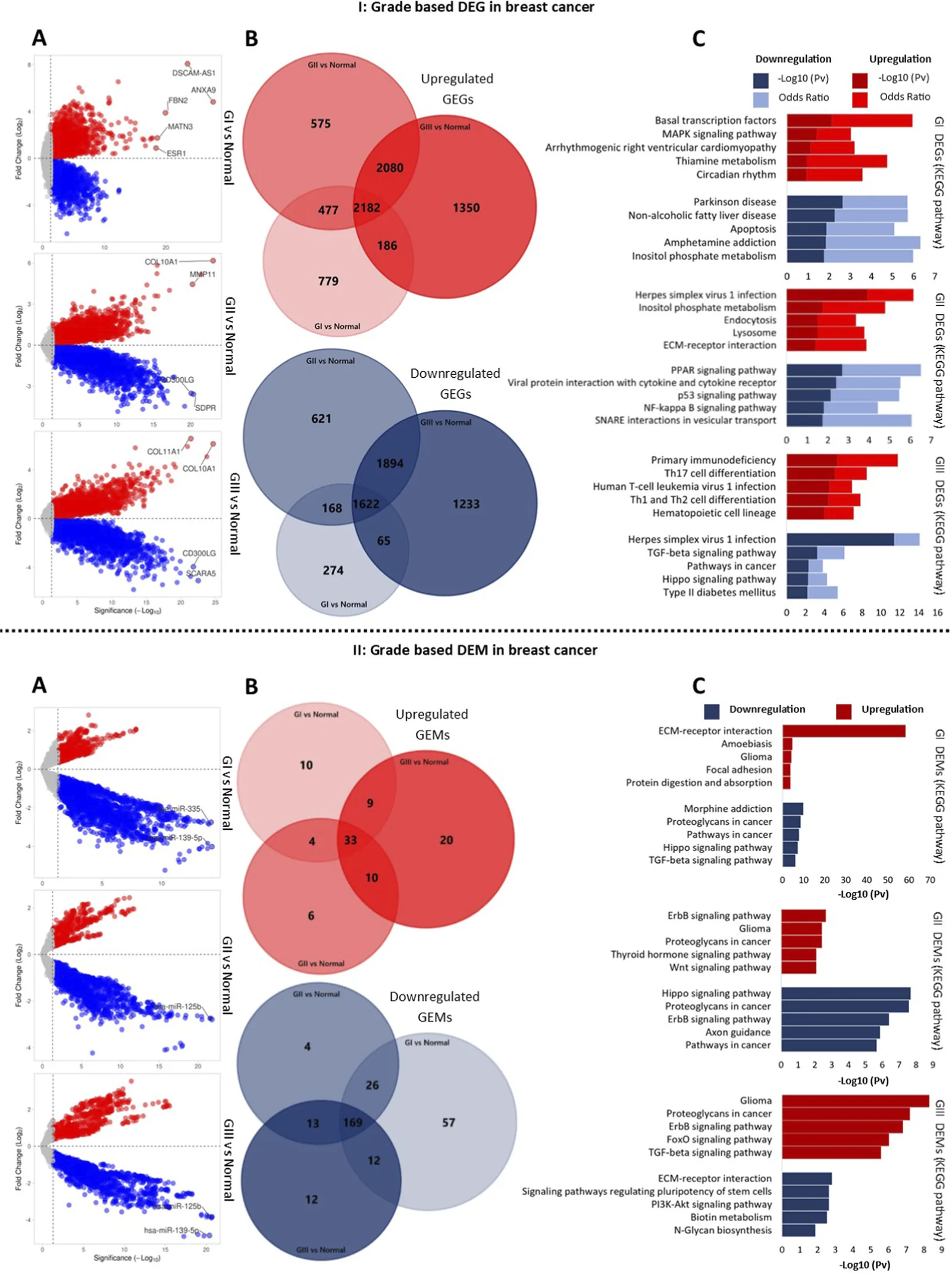
Data-based bioinformatic analysis of **(I)** mRNA (GSE29044) expression and **(II)** miRNA (GSE45666) in breast tumors. **(IA,IIA)** Volcano plot of mRNA and miRNA expression in GI, GII, and GIII breast tumors compared to normal tissue. **(IB,IIB)** Venn diagram of overlap of upregulated (red) and downregulated (blue) factors in three breast cancer grades. **(IC,IIC)** KEGG pathway enrichment analysis of upregulated (red) and downregulated (blue) mRNAs and miRNAs in GI, GII, and GIII breast cancer grades. Bars show -log10 (P-value) and odds ratio.

The analysis of DEGs and DEMs showed significant differences in their expression levels among low-grade (GI), intermediate-grade (GII), and high-grade (GIII) breast tumors compared to normal breast tissue (Figure 1). The upregulated and downregulated DEGs and DEMs for each grade are summarized in Supplementary Material 2.

The volcano plot and Venn diagram indicated an increase in both the variation and expression levels of mRNAs with increasing tumor grade (Figures 1IA,IB). A total of 779 mRNAs in GI tumors, 575 mRNAs in GII tumors, and 1350 mRNAs in GIII tumors were upregulated, while 274 mRNAs in GI tumors, 621 mRNAs in GII tumors, and 1234 mRNAs in GIII tumors were downregulated (Figure 1IB). KEGG pathway analysis revealed that different pathways were associated with different grades of breast carcinoma.

The major pathways associated with the 779 upregulated DEGs in GI tumors were basal transcription, MAPK signaling, and arrhythmogenic right ventricular cardiomyopathy (Figure 1IC). On the other hand, Parkinson’s disease, non-alcoholic fatty liver disease, and apoptosis were the most suppressed pathways associated with the 272 downregulated mRNAs in GI tumors (Figure 1IC). The KEGG enrichment results of the 575 upregulated mRNAs in GII tumors showed that herpes simplex virus 1 infection, inositol phosphate metabolism, and endocytosis were the major mechanisms, whereas the PPAR pathway, viral protein interaction with cytokine and cytokine receptor, and p53 signaling pathway were associated with the 621 downregulated mRNAs in GII tumors (Figure 1IC). The 1350 upregulated DEGs in GIII tumors were associated with primary immunodeficiency, Th17 cell differentiation, and human T-cell leukemia virus 1 infection, whereas the signaling pathways of herpes simplex virus 1 infection, TGF-β signaling, and pathways in cancer were the most affected among the 1234 downregulated DEGs in GIII tumors (Figure 1IC).

Figures 1IIA,IIB show that the volcano plot and Venn diagram revealed a higher degree of downregulation among the DEMs in all three bast carcinoma grades. In particular, 10 miRNAs were upregulated in GI, 6 in grade II GII, and 20 in GIII breast carcinomas, while 56, 55, and 12 miRNAs were downregulated in GI, GII, and GIII tumors, respectively (Figure 1IIB). KEGG enrichment analysis of the 10 upregulated miRNAs in GI tumors showed significant involvement of pathways related to ECM-receptor interaction (P = 4.55E-59), amoebiasis (P = 2.79E-05), and glioma (P = 8.46E-05). The analysis of the 56 downregulated miRNAs in GI tumors indicated significant association with pathways involved in morphine addiction (P = 1.58E-10), proteoglycans in cancer (P = 2.94E-09), and pathways in cancer (P = 1.93E-08). In GII tumors, the ErbB signaling pathway (P = 0.002), proteoglycans in cancer (P = 0.004), and glioma (P = 0.004) were the three most significant cascades associated with the 6 upregulated miRNAs. The Hippo pathway (P = 2.14E-08), proteoglycans in cancer (P = 2.65E-08), and ErbB signaling pathway (P = 4.05E-07) were the major signaling pathways associated with the 55 downregulated miRNAs in GII tumors (Figure 1IIC). Additionally, the 20 upregulated miRNAs in GIII tumors were associated with activation of pathways related to glioma (P = 4.99E-09), proteoglycans in cancer (P = 6.15E-08), and the ErbB signaling pathway (P = 1.55E-07) (Figure 1IIC). Finally, the 12 downregulated miRNAs in GIII tumors showed significant downregulation of pathways related to ECM-receptor interaction (P = 0.0016), PI3K-Akt signaling pathway (P = 0.0024), and signaling pathways regulating pluripotency of stem cells (P = 0.0024) (Figure 1IIC).

### 3.2 MGD pathways and PPI in breast carcinoma progression

**FIGURE 2:**
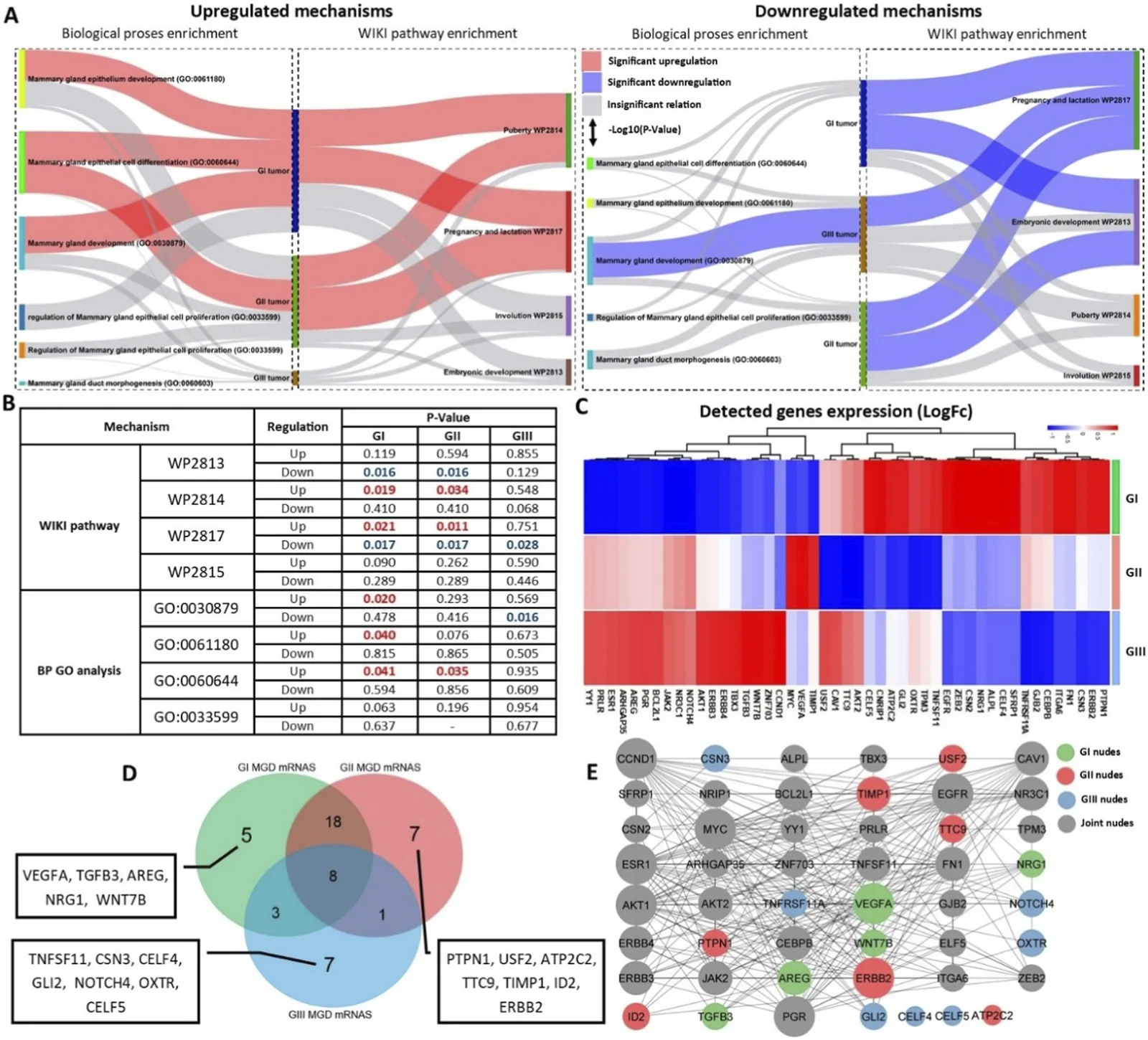
Mechanisms of mammary gland development and miRNA expression in breast cancer grades. **(A)** Sankey diagram illustrating the contribution of mammary gland development (MGD) stages in GI, GII, and GIII breast cancer grades. The red graph represents upregulated DGEs, while the blue graph represents downregulated DGEs. Red and blue ribbons show significant correlation, and gray bands indicate insignificant correlation. The width of each ribbon represents the -log10 (P-value). **(B)** Table of the P-value level of detected mechanisms in each breast cancer grade. Numbers in red indicate significantly upregulated mechanisms, while numbers in blue indicate significantly downregulated mechanisms. **(C)** Heatmap showing the expression level of target mRNAs in breast cancer grades compared to normal tissue, based on LogFC. **(D)** Venn diagram of the overlap of MGD mRNAs in three grades of breast cancer. **(E)** Protein-protein interaction network of MGD mRNAs in three grades of breast cancer. The size of nodes reflects the level of interaction, and the color of nodes refers to the mRNA group.

The results of the WIKI pathways and GO analysis of the DEGs in low, moderate, and high-grade breast tumors revealed that the MGD process was significantly engaged in the lower-grade tumors (GI and GII). Our analysis showed that this pattern became diluted as the breast cancer grade increased. Our results showed that the MGD stages of puberty and pregnancy/lactation were significantly associated with the upregulated DEGs in both GI and GII tumors (Figures 2A,B). Additionally, the embryonic development stage was found to be significantly related to both upregulated and downregulated DEGs in both GI and GII tumors. The only stage found to be significantly downregulated by MGD in GIII tumors was pregnancy/lactation (Figures 2A,B).

The overrepresentation analysis of the biological processes of the upregulated DEGs in GI tumors revealed significant involvement in mammary gland epithelium development, mammary gland epithelial cell differentiation, and mammary gland development mechanisms (Figures 2A,B). However, no significant MGD mechanism was detected in the downregulated DEGs in GI tumors. In GII tumors, only mammary gland epithelium development was found in the upregulated gene lists, with no significant link detected in the downregulated DEGs. The only significant MGD mechanism found in the downregulated genes in GIII tumors was mammary gland development (Figures 2A,B).

The expression changes (LogFc) of 49 target mRNAs were visualized in the three grades of breast cancer. Venn analysis revealed 5 specific genes in GI, 7 in GII, and 7 in GIII tumors (Figures 2C,D). The PPI analysis of these 49 mRNAs showed that the GI and GII tumor-specific mRNAs play a central role in the network (Figures 2D,E). The hub genes with the highest interaction degree were ESR1 (33 edge count), AKT1 (31 edge count), MYC (30 edge count), ERBB2 (27 edge count), EGFR (26 edge count), CCND1 (26 edge count), and PGR (22 edge count). The GI tumor-specific mRNAs AREG and TIMP1 also had high interaction degrees, with 13 and 11 edge values, respectively. The mRNAs TP2C2 from GII and CELF4/CELF5 from GIII tumors were not involved in this network (Figure 2E).

### 3.3 miRNA–mRNA network analysis and candidate miRNA enrichment

**FIGURE 3:**
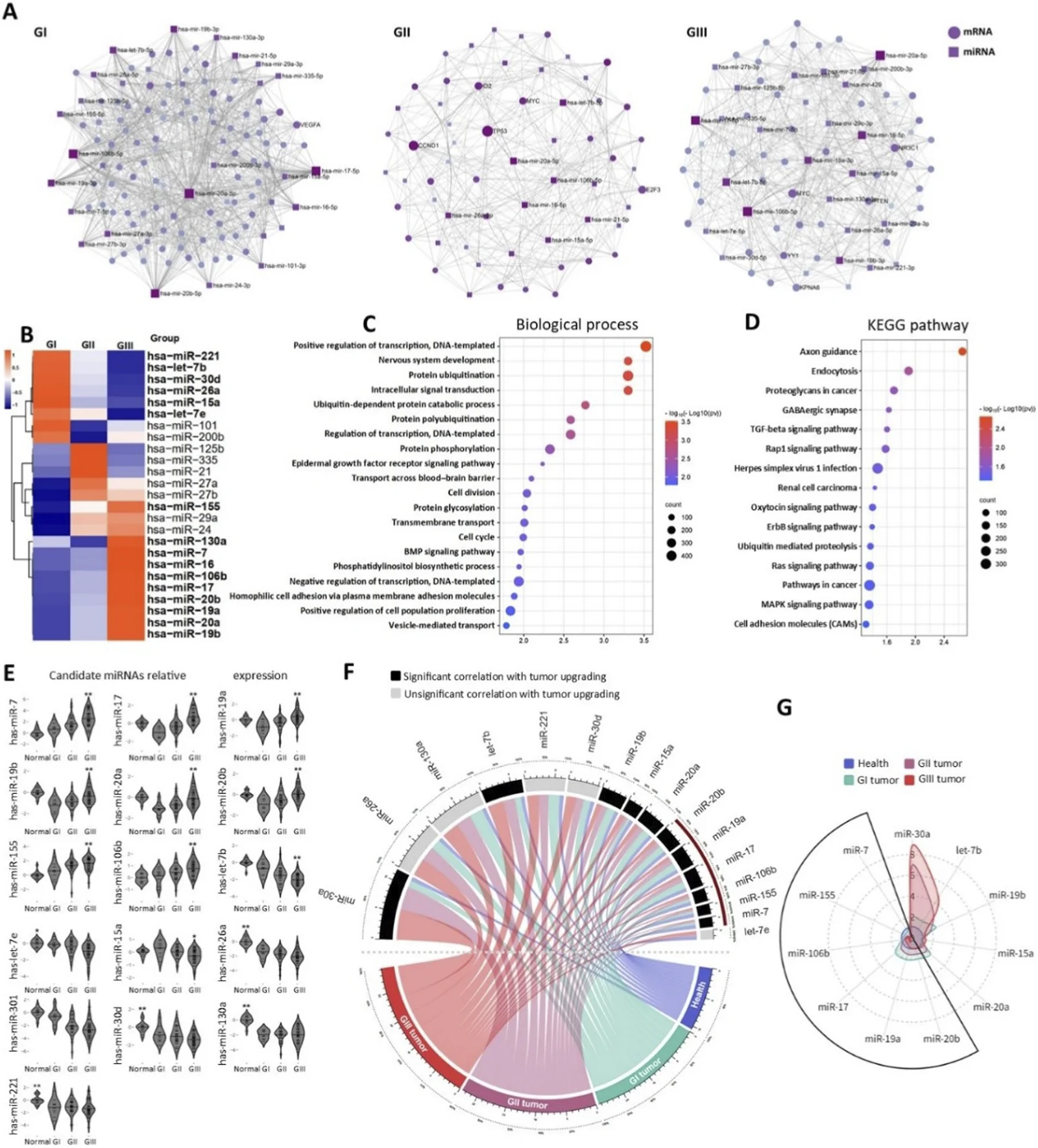
Analysis of miRNA-mRNA network, expression patterns in different grades of breast cancer, and enrichment of target miRNAs. **(A)** The miRNA-mRNA interaction network in GI, GII, and GIII breast cancer is shown, with square nodes representing miRNAs and circular nodes representing mRNAs. The size and color sharpness of nodes indicate the highest interacting nodes. **(B)** The heatmap shows the expression levels of miRNAs in three different breast tumor grades compared to normal tissue, based on log2 fold change (log2FC). The red color represents upregulation, while the blue color represents downregulation. **(C)** Biological process enrichment in GO terms in eleven candidate miRNAs is shown. **(D)** KEGG pathway enrichment in eleven candidate miRNAs is shown. **(E)** The violin plot shows the relative expression levels (ΔΔCt) of 16 candidate genes using GSE26659 samples. **(F)** The circular plot for the visualization of the averaged (mean) 2^−ΔΔCT^ expression of 16 miRNAs in four groups. Black rectangles indicate miRNAs with a significant correlation with tumor upgrading, and gray rectangles indicate miRNAs with an insignificant correlation. The red rectangle indicates the six target miRNAs with a gain-of-function pattern. **(G)** The radar plot shows the averaged (mean) 2^−ΔΔCT^ expression of 11 significantly detected miRNAs in four groups.

A different miRNA-mRNA interaction pattern was found in the GI, GII, and GIII tumors MGD mRNAs, with significantly altered expressed miRNAs (Figure 3A). The GI tumor showed a more complex miRNA-mRNA interaction network with more interacting nodes, with 22 miRNAs involved, and hsa-miR-17-5p, hsa-miR-20a-5p, hsa-miR-20b-5p, and hsa-miR-106-5p being the hub miRNAs. VEGFA was the most involved mRNA in the GI tumor-specific interaction network. The GII tumor miRNA-mRNA interaction network had the lowest degree of interaction, with only 7 potential miRNAs, including hsa-let-7b-5p, hsa-miR-15a-5p, hsa-miR-16a-5p, hsa-miR-20a-5p, hsa-miR-21-5p, hsa-miR-26a-5p, and hsa-miR-106b-5p, detected as hub miRNAs. TP53, CCND1, and MYC were the primary MGD mRNAs in the GII tumor miRNA-mRNA network. In the GIII tumor, 23 miRNAs with high interaction were detected, with hsa-miR-20a-5p, hsa-miR-17-5p, hsa-miR-16-5p, and hsa-miR-106b-5p interacting most frequently. NB3C1, PTEN, MYC, YY1, and KPNA6 mRNAs were the major MGD mRNAs of the GIII tumor (Figure 3A).

After the miRNA-mRNA interaction analysis, 25 miRNAs were selected as targets of interest (Figure 3B). The heatmap results for miRNA expression in the GI, GII, and GIII tumors from the GSE45666 DEMs showed that 10 microRNAs had a sharp gain-of-function expression pattern in the stem tumor compared with the low-grade tumor, including miR-7, miR-16, miR-17, miR-19a, miR-19b, miR-20b, miR-20b, miR-106 b, miR-130a, and miR-155. In contrast, let-7b, let-7e, miR-15a, miR-26a, miR-30d and miR-221 had a sharp loss-of-function expression pattern. The enrichment analysis of the 10 candidate miRNAs with gain-of-function revealed involvement in the following biological processes positive regulation of transcription, DNA-templated (P = 0.0003), nervous system development (P = 0.0005), protein ubiquiti-nation (P = 0.0005), intracellular signal transduction (P = 0.0017), and ubiquitin-dependent protein cata-bolic process (P = 0.002) (Figure 3C). These miRNAs were also enriched in KEGG pathways, including ax-on guidance (P = 0.002), endocytosis (P = 0.012), proteoglycans in cancer (P = 0.02), GABAergic syn-apse (P = 0.023), and TGF-β signaling pathway (P = 0.025) (Figure 3D).

Through analyzing the relative expression of the total 16 candidate miRNAs, we discovered that miRNAs 7, 17, 19a, 19b, 20b, 155, and 106b were significantly upregulated (P < 0.05) in GIII tumors compared to lower-grade tumors (GI and GII) and healthy groups (Figure 3E). Conversely, let-7b, miR-15a, and miR-30a were significantly downregulated (P < 0.05) in GIII tumors compared to the lower grade tumors and healthy groups (Figure 3E). Furthermore, we observed a significant upregulation (P < 0.05) of let-7e, miR-26a, miR-30d, miR-13a, and miR-221 in normal breast tissue compared to breast tumors (Figure 3E). Based on the correlation circular plot (Figure 3F) and radar chart (Figure 3G), which display the expression correlation (2^−ΔΔCT^ scale) of the significantly expressed miRNAs in GIII tumors compared with GI and GII tumors, we selected miRNAs 7, 17, 19a, 20b, 106b, and 155 as potential miRNAs with a gain-of-function pattern for the experimental phase.

### 3.4 miRNAs qRT-PCR validation and prognostic network analysis

**FIGURE 4:**
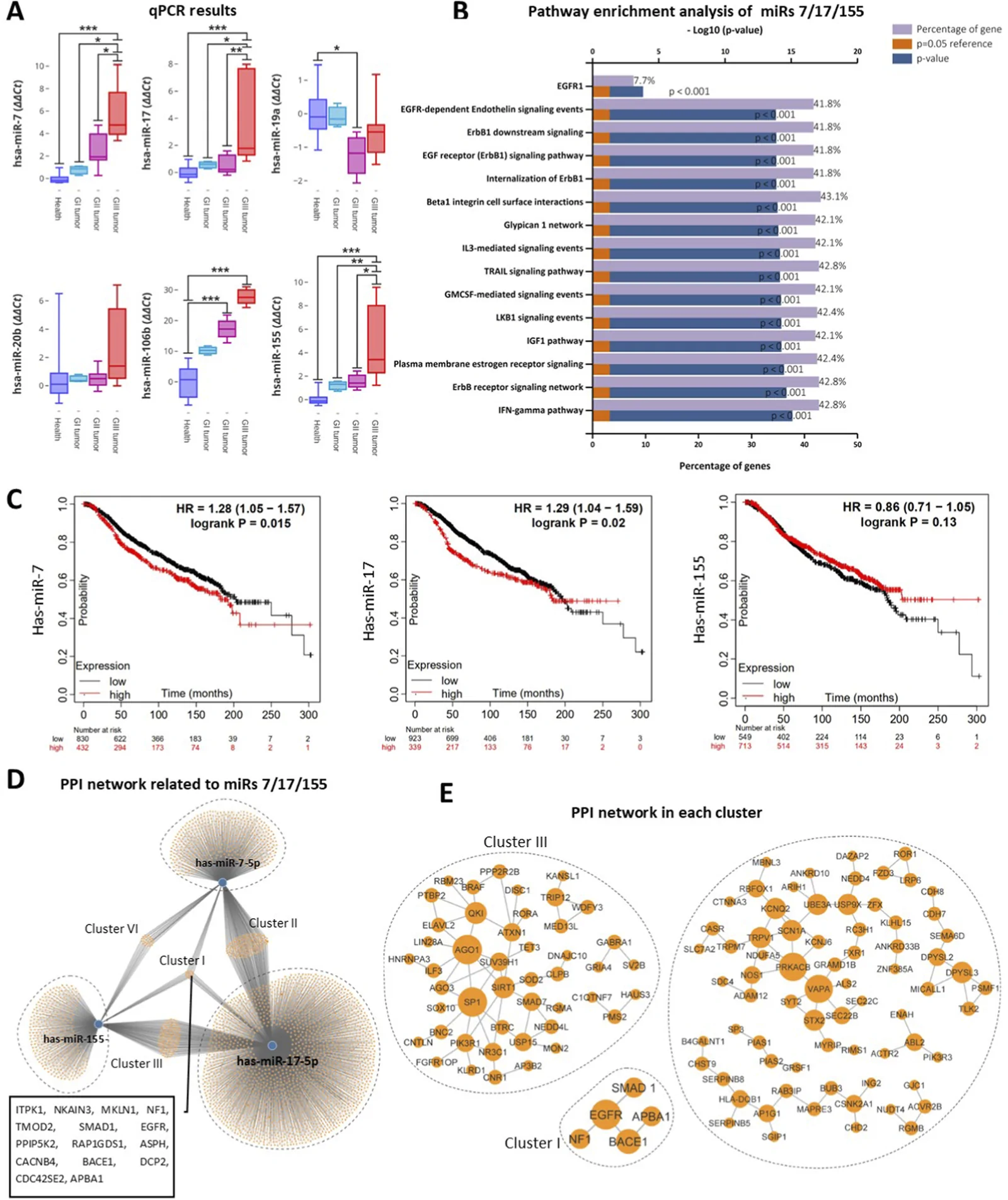
qRT-PCR analysis of target miRNAs and their involved mRNA interaction network. **(A)** Boxplots showing the expression of miR-7, miR-17, miR-19a, miR-20b, 106b, and miR-155 in normal tissues and in grades I, II, and III tumors, normalized using the *ΔΔCt* method. **(B)** Pathways enriched in miRNAs with significant changes in expression. **(C)** Kaplan-Meier survival curve of breast cancer patients based on the expression levels of miR-7, miR-17, and miR-155, as obtained from the online Kaplan-Meier plotter database. **(D)** Network of interactions between target miRNAs and mRNAs, with cluster I representing intermediate miRNAs among the three miRNAs, cluster II representing shared mRNAs between miR-7 and miR-17, cluster III representing shared mRNAs between miR-17 and miR-155, and cluster VI representing shared mRNAs between miR-155 and miR-7. **(E)** PPI network of different clusters, with node size indicating interaction level.

The expression of the target miRNAs was analyzed in tumor and normal tissue samples from 27 patients using qPCR. The clinicopathological characteristics of the patients are summarized in the Supplementary Table S1. The results of the qPCR analysis showed that miR-7, miR-17, and miR-155 were significantly upregulated in GIII tumors compared to normal tissue, GI tumors, and GII tumors (Figure 4A). We also detected a relevant upregulation pattern of these miRNAs associated with increased tumor stemness. The average expression levels of miR-7 were −0.013 in normal tissue, 0.741 in GI tumors, 2.510 in GII tumors, and 5.973 in GIII tumors. The significance coefficients (P-value) between GIII tumors and normal tissue, GI tumors, and GII tumors were calculated to be 0.00, 0.02, and 0.05, respectively (Figure 4A). Similarly, miR-17 expression levels were 0.00 in normal tissue, 0.554 in GI tumors, 0.604 in GII tumors, and 4.033 in GIII tumors. The P values between GIII tumors and normal tissue, GI tumors, and GII tumors were determined to be 0.00, 0.026, and 0.03, respectively (Figure 4A).

Furthermore, miR-155 expression levels were 0.005 in normal tissue, 1.195 in GI tumors, 1.556 in GII tumors, and 4.663 in GIII tumors. The significance coefficients between GIII tumors and normal tissue, GI tumors, and GII tumors were calculated to be 0.00, 0.019, and 0.05, respectively (Figure 4A). However, no significant upregulation of miR-19a, miR-20b, and miR-106b was detected in GIII breast cancer compared to GI and GII tumors. The expression pattern of the miRNAs in the four groups is summarized in Figure 4A.

Our pathway enrichment analysis revealed that miRs 7/17/155 predominantly regulate EGFR-associated signaling pathways in breast cancer. Furthermore, interferon gamma (IFN-γ), plasma membrane receptor (PMR) signaling, insulin-like growth factor 1 (IGF1), inhibitor of nuclear factor kappa B kinase complex (IKB1), and granulocyte-macrophage colony-stimulating factor (GMCSF) mediated signaling events were identified as other related pathways to miRs 7/17/155 (Figure 4B). The survival analysis demonstrated a significant association between expression levels of mir-7 and mir-177 and the survival of breast cancer patients with p-values of 0.015 and 0.02 and hazard ratios of 1.28 and 1.29, respectively (Figure 4C). However, the expression level of mir-155 did not show a significant impact on patient survival (p = 0.13) (Figure 4C).

Using the miRWalk database, we found that the 3′-UTR regions of 1219, 4762, and 946 mRNAs are complementary to hsa-miR-7-5p, hsa-miR-17-5p, and hsa-miR-155-5p, respectively, with scores ≤95.15 mRNAs were identified in the intersection network of these mRNA sets, including ITPK1, NKAIN3, MKLN1, NF1, TMOD2, SMAD2, EGFR, PIP5K2, RAP1GDS1, ASPH, CACNB4, BACE1, DCP2, CDC42SE2, and APBA1 (Figure 4D). The PPI analysis of this cluster revealed a network composed of the interactions between EGFR/SMAD1/NF1/BACE1/APBA1 (Figure 4E). There were 143 common mRNAs between the target mRNAs of miR-7 and miR-17. The PPI analysis of Cluster II showed a network consisting of 46 nodes and 56 edges, with AGO1 and SP1 having the highest number of interactions (Figure 4E). Cluster III, consisting of 123 common mRNAs between miR-17 and miR-155, had a PPI network composed of 72 nodes and 73 edges. PRKACB and VAPA were identified as the hub mRNAs in this PPI network. Cluster IV, consisting of 26 shared mRNAs between miR-17 and miR-155, did not show a remarkable interaction network (Figure 4E).

### 3.5 EGFR-mediated EMT patterns and correlations analyses

**FIGURE 5:**
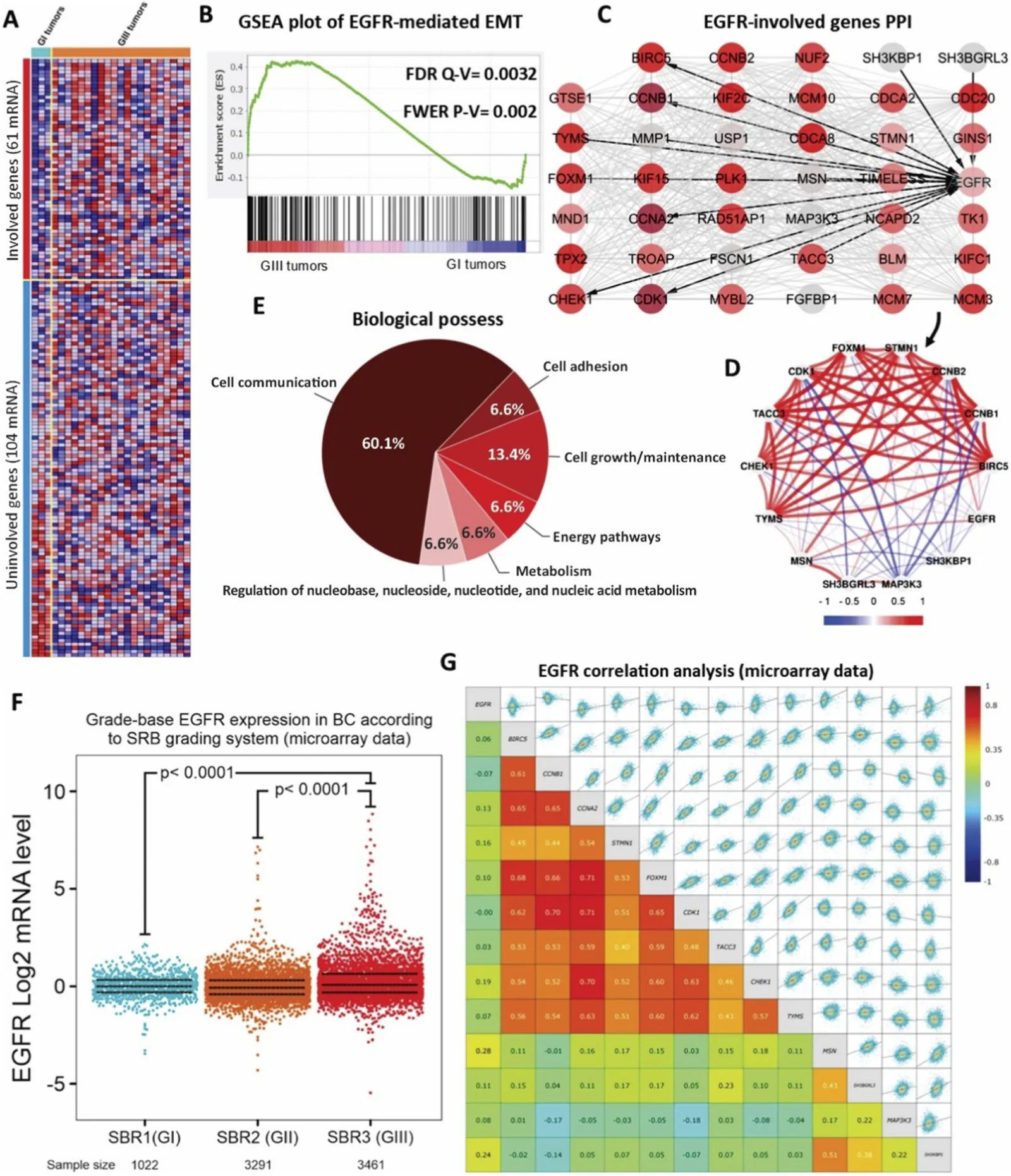
Tracking EGFR-mediated EMT in high-grade breast tumors. **(A,B)** GSEA results of samples from the GSE29044 data file in grade I and grade III tumors. **(C)** PPI network of EGFR with 61 genes involved in EGFR-EMT in grade III tumors. **(D)** Correlation coefficient network diagrams for mRNAs involved in EGFR-mediated epithelial-mesenchymal transition and their interaction with EGFR. **(E)** Results of the analysis of the biological properties of mRNAs interacting with EGFR. **(F)** Expression level of EGFR mRNA in different grades of breast cancer using the breast carcinoma-GenExMiner 4.5 expression model. **(G)** Correlation analysis of EGFR with the 13 most highly interacting mRNAs using the breast carcinoma-GenExMiner 3.0 correlation analysis model.

The results of our GSEA analysis showed that the EGFR-mediated EMT mechanism is significantly more active in GIII tumors than in GI tumors (FWER P = 0.002). To establish this, we evaluated the expression levels of 165 genes involved in the EGFR-mediated EMT gene set. Our analysis revealed that 64 of these genes were upregulated in GIII tumors and downregulated in GI tumors (Figures 5A,B).

Our PPI analysis of the 64 EGFR-mediated EMT genes found in GIII tumors identified 13 key genes (BIRC5, CCNB1, CCNB2, STMN1, FOXM1, CDK1, TACC3, CHEK1, TYMS, MSN, SH3BGRL3, MAP3K3, and SH3KBP1) that interact with EGFR (Figure 5C). Among these, CCNA2 (degree = 33), CCNB1 (degree = 32), and CDK1 (degree = 32) were the most highly interacting nodes and directly related to EGFR (Figure 5C). The correlation analysis of the expression results of 14 genes in 21 GIII tumor samples showed a significant positive correlation between the expression levels of EGFR and BIRC5 (r = 0.60, p < 0.05), CCNB1 (r = 0.69, p < 0.01), CCNB2 (r = 0.61, p < 0.05), and STMN1 (r = 0.52, p < 0.05). This indicates that the expression of the EGFR gene is positively related to the expression of BIRC5, CCNB1, CCNB2, and STMN1 genes in GIII tumors. There were no significant correlations between the expression levels of EGFR and other genes such as FOXM1, CDK1, TACC3, CHEK1, TYMS, and others (Figure 5C).

The results of the coefficient analysis showed significant positive correlations between EGFR and BIRC5 (r = 0.717, P < 0.05), CCNB1 (r = 0.696, p < 0.05), CCNB2 (r = 0.831, p < 0.05), and STMN1 (r = 0.706, P < 0.05), suggesting that the expression levels of these genes are positively associated with the expression of EGFR in GIII tumor samples. Insignificant correlations were observed between EGFR and FOXM1 (r = −0.217, p > 0.05), CDK1 (r = 0.187, p > 0.05), TACC3 (r = 0.146, p > 0.05), CHEK1 (r = −0.252, p > 0.05), and other genes. A significant positive correlation was also observed between CCNB1 and CCNB2 (r = 0.938, p < 0.05), indicating a strong association between the expression of these two genes in GIII tumor samples (Figure 5D). Biological process enrichment of the EGFR-correlated genes in high-grade stem tumors showed major involvement in cell communication (60.1%), cell growth and/or maintenance (13.4%), and smaller proportions in cell adhesion (6.6%), energy pathways (6.6%), metabolism (6.6%), and nucleic acid metabolism (6.6%) (Figure 5E). All these processes are associated with EMT in breast cancer.

In the gene expression analysis conducted using the Breast carcinoma-GenExMiner v4.5 database, the results showed that EGFR is expressed at a significantly higher level in GIII tumors compared to both GI and GII tumors (p < 0.0001). The mean and median EGFR expression levels in GIII tumors were also found to be the highest among the three tumor types (Figure 5F). Additionally, the correlation analysis of 14 genes in breast cancer using the Breast carcinoma-GenExMiner v4.5 database revealed that EGFR gene has a moderate positive correlation with several other genes, including TYMS (r = 0.19), MSN (r = 0.28), SH3BGRL3 (r = 0.24), BIRC5 (r = 0.06), and others, with all correlations being significant (p < 0.0001) (Figure 5G). The analysis also showed that EGFR has a low negative correlation with the CCNB1 gene (r = −0.07, p < 0.0001). Furthermore, the analysis revealed that a cluster of nine genes, including BIRC5, CCNB1, CCNA2, STMN1, FOXM1, CDK1, TACC3, CHEK1, and TYMS, had the highest correlation in breast cancer (Figure 5G). As the outcome of this part, the breast carcinoma-GenExMiner 3.0 correlation analysis confirmed that EGFR expression in GIII tumors is consistently associated with genes such as BIRC5, CCNB1, and STMN1, while showing weak or negative associations with FOXM1, CDK1, TACC3, and CHEK1, in agreement with our GSEA-based findings (Figures 5D,G).

### 3.6 EGFR expression via IHC and its prognostic significance

**FIGURE 6:**
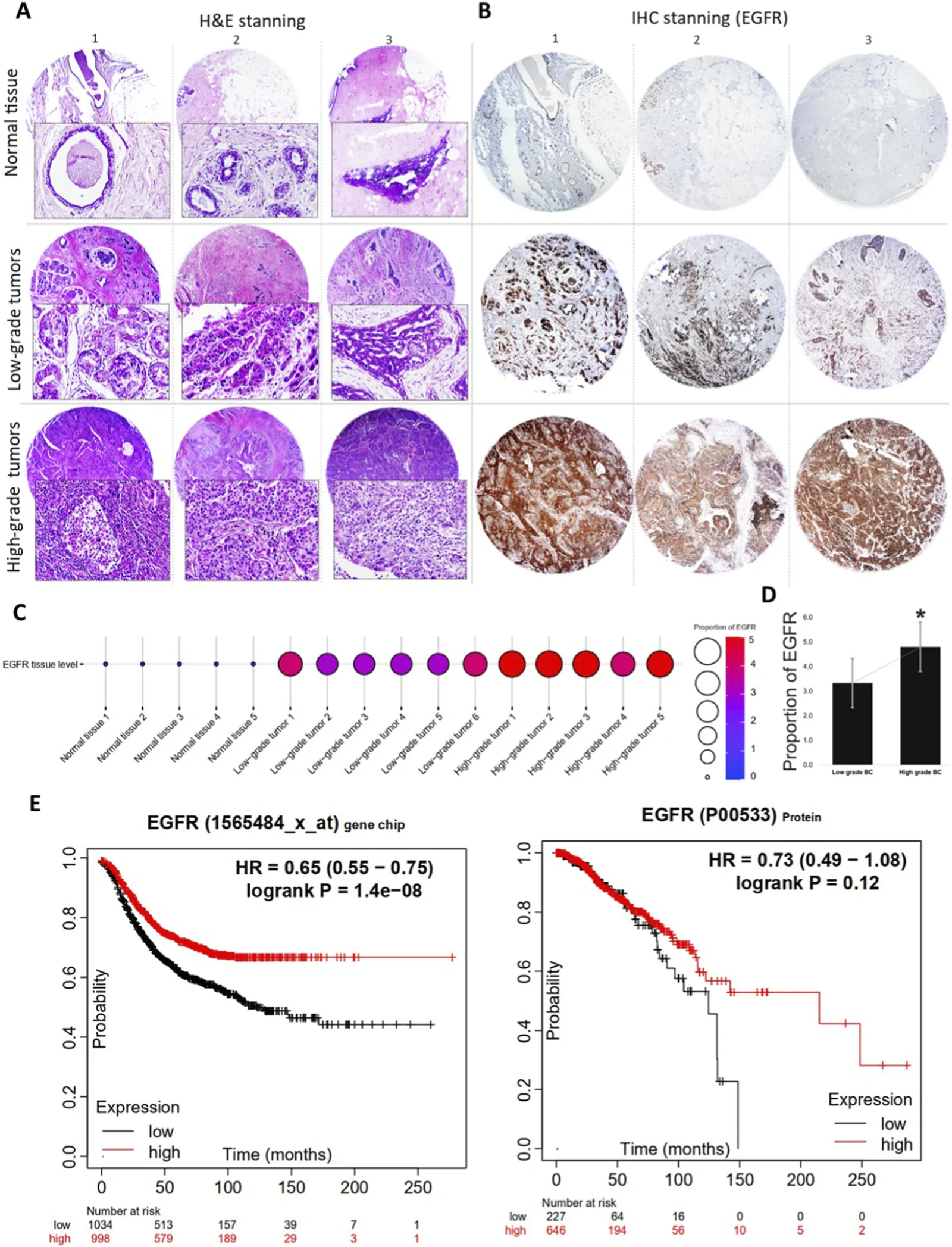
Tissue microarray-based IHC analysis and survival analysis in GI and GIII tumors. **(A)** H&E staining of normal tissue, GI, and GIII tumors (4× and 40× magnification). **(B)** EGFR + cell levels as assessed by IHC staining in normal tissues, GI, and GIII tumors (4× magnification). **(C)** Balloon plot displaying EGFR histological levels in normal tissues, GI, and GIII tumors. **(D)** Bar graph showing EGFR histological levels in normal tissues and GI and GIII tumors. **(E)** Kaplan-Meier survival curve of breast cancer patients based on EGFR mRNA and protein expression level, as obtained from the online Kaplan-Meier plotter database.

The MTA analysis was performed on tumor and normal tissue samples from 11 patients. Supplementary Table S3 provides a summary of the clinicopathological characteristics of the patients. The histological grade of tissue samples was evaluated using H&E staining (Figure 6A). IHC analysis showed significantly higher EGFR-positive cells in undifferentiated breast tumors compared to normal breast tissue and well-differentiated breast cancer (GI), as demonstrated in Figure 6B. EGFR expression levels significantly differed among the normal tissue, well-differentiated tumor, and undifferentiated tumor (GIII) groups. Pair-wise comparisons showed a significant difference in EGFR expression levels between normal tissue and GIII tumor (p = 0), no significant difference between normal tissue and GI tumor (p = 0.065), and a significant difference between GI and GIII tumors (p = 0.05) (Figures 6C,D). The average EGFR expression levels were 0 in normal tissue, 3.33 in well-differentiated tumors, and 4.48 in undifferentiated tumors.

The Kaplan-Meier plotter database analysis revealed a significant association between EGFR mRNA expression levels and patient survival in breast cancer patients (Figure 6E). The analysis showed that increased EGFR mRNA expression levels were associated with lower patient survival, as evidenced by a hazard ratio (HR) of 0.65 for the gene chip method and 1.35 for the RNA sequencing method. However, no significant association was found between EGFR protein expression levels and patient survival (Figure 6E).

### 3.7 ScRNAseq analysis

**FIGURE 7:**
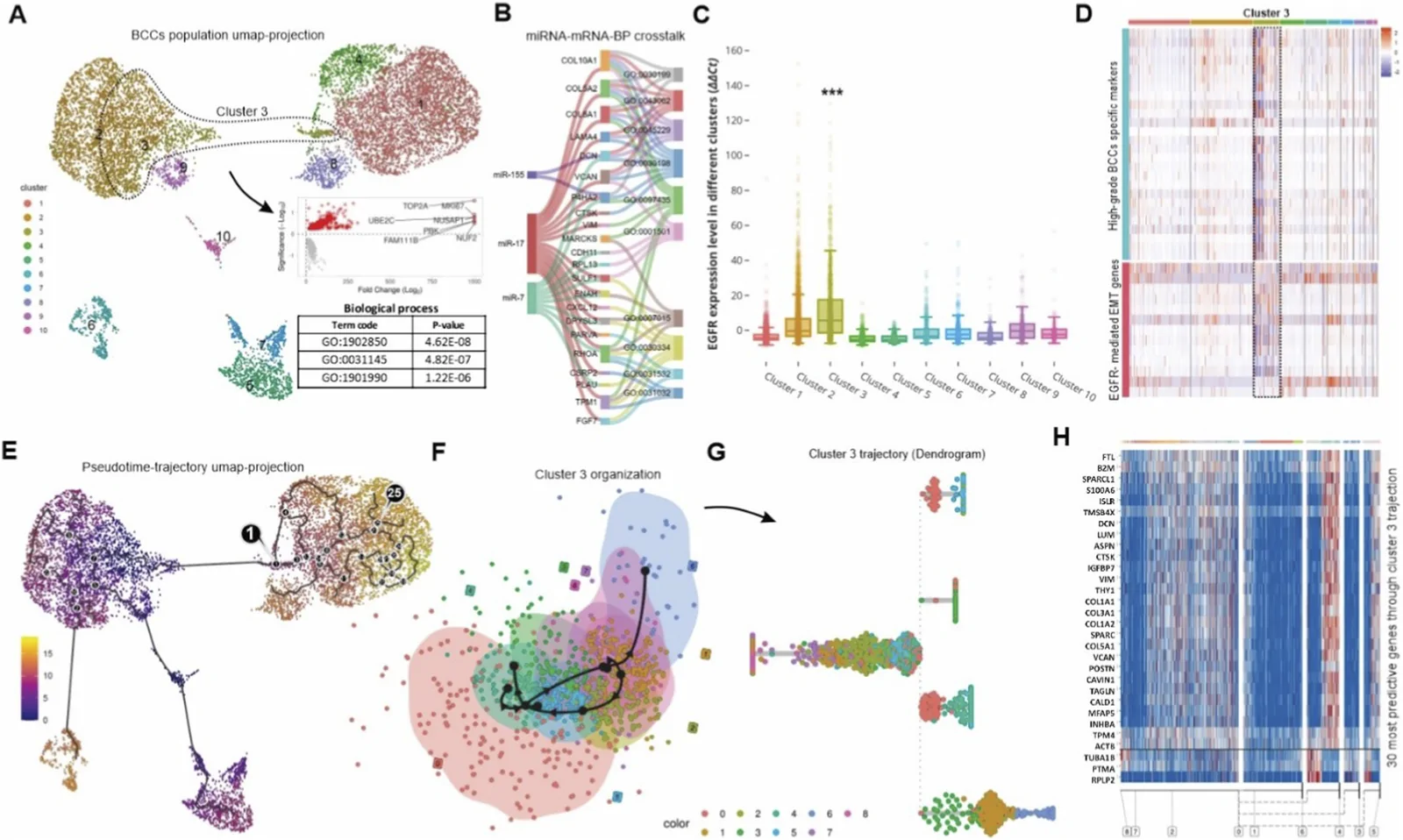
Single-cell RNA sequencing analysis to track EGFR-mediated EMT on the undifferentiated breast tumor cellular population and pseudotime trajectory analysis. **(A)** UMAP plot that annotates ten major cell types in a breast tumor. The volcano plot represents the expression pattern of cluster 3 DEGs. The table presents the results of biological process enrichment analysis for cluster 3 upregulated DEGs. **(B)** Sankey diagram illustrating the crosstalk between miRNA cluster 7/17155, cluster 3 upregulated DEGs, and biological process terms regulated by the genes. **(C)** Box plot showing the expression of EGFR in ten cellular populations of breast cancer using the ΔΔCt method. **(D)** Heatmap representing expression of high-grade tumors of breast-specific markers and target EGFR-mediated EMT genes between the ten clusters of breast cancer cells. The red color represents upregulation, while the blue color represents downregulation. **(E)** Pseudotime trajectory UMAP projection of the ten major cell clusters of breast tumor. **(F,G)** Cluster 3 cell organization and trajectory analysis. The analysis shows the classification of eight main cellular populations within cluster 3. **(H)** Heatmap representing the expression of 30 main markers throughout cluster 3 organization. The red color represents upregulation, while the blue color represents downregulation.

After the removal of low-quality cells, a total of 10,602 cells were included in the analysis. Of these cells, 53.9% were detected in the G1 phase of mitosis, 28.6% were detected in S phase, and 17.6% were detected in G2M phase (Supplementary Materials 2, 3). Using the Seurat package in R, these cells were classified into ten main cell clusters (C1-C10) (Supplementary Figures S1A,F). Detection of specific stem breast tumor markers (Supplementary Material 2) revealed that C3 represented the high-grade cellular population, which was the target of our EGFR-mediated EMT genes (SH3BGRL3, SH3KBP1, BIRC5, CCNB1, CCNB2, STMN1, FOXM1, CDK1, TACC3, CHEK1, TYMS, MSN, and MAP3K3), showing a strong association with this cluster (Figure 7E).

Besides, our analysis of EGFR expression in the cells from all ten populations demonstrated that EGFR was dramatically upregulated in C3 cells compared to the other clusters. On average, C3 showed the highest EGFR expression level, with a value of 12.25. Clusters 1, 4, 5, 7, and 8 had lower expression levels, ranging from −4.73 to 3.04, while clusters 2, 6, 9, and 10 had intermediate expression levels, ranging from −0.77 to 4.04 (Figure 7C). Statistical analysis indicated that C3 had a significantly higher EGFR expression level compared to all other clusters, with a p-value of 0. This suggests that cluster 3 has a distinct molecular signature characterized by elevated EGFR expression. Conversely, clusters 1, 4, 5, 7, and 8 had significantly lower EGFR expression levels compared to cluster 3, with p-values ranging from 0.034 to 0.051 (Figure 7C).

Enrichment analysis of the main DEGs in C3 with an average log2FC > 1 revealed significant involvement in several biological processes. The main enriched mechanisms included microtubule cytoskeleton organization involved in mitosis (GO:1902850), anaphase-promoting complex-dependent catabolic process (GO:0031145), and regulation of mitotic cell cycle phase transition (GO:1901990) (Figure 7A). Based on the snaky plot analysis of miRNA cluster 7/17/155 with the C3 upregulated DEGs and biological processes regulating EMT, we found several correlations. MicroRNA-155 was observed to target DCN and P4HA2, while miR-17 targeted COL10A1, COL5A2, COL8A1, CSRP2, CTSK, CXCL12, DPYSL3, ENAH, FGF7, LAMA4, PARVA, RHOA, SULF1, TPM1, VCAN, and VIM. Additionally, miR-7 targeted CDH11, DCN, DPYSL3, ENAH, P4HA2, PLAU, RHOA, and RPL13. GO analysis revealed that VCAN, P4HA2, CTSK, LAMA4, COL5A2, COL10A1, COL8A1, and DCN were significantly enriched in the biological process GO terms, including “positive regulation of extracellular matrix organization”, “collagen fibril organization”, “cartilage development”, and “cell adhesion” (Figure 7B).

Utilizing monocle3, the trajectory pseudotime UMAP projection displayed that the C3, which was identified as the undifferentiated population in breast carcinoma cells, constituted the initial cluster in the cellular population. Additionally, this analysis revealed that the other cellular subtypes were developed from this cluster, indicating its critical role in the development and differentiation of these cells (Figure 7E). Using Asc-Seurat on C3, we detected that this undifferentiated malignant population was generated from nine niches (N0-N8). Our analysis revealed that N0 was likely the major population responsible for the development of additional C3 cells, as indicated in Figures 7F,G. Furthermore, we compiled a list of the most important genes within the trajectory of cluster 3, which can be found in the Supplementary Material 2. Notably, trajectory analysis highlighted 30 predictive genes, such as ECM-related (COL1A1, COL3A1, SPARC, POSTN), cytoskeletal (VIM, ACTB, TUBA1B), and regulatory genes (IGFBP7, B2M, INHBA), that characterize the progression of the breast carcinomas’ cluster 3 populations and are closely linked to stemness features and EMT regulation (Figure 7H).

## 4 Discussion

Developmental biology perspective can provide a useful insight to the mechanisms of tumor upgrading and the formation of high-grade and undifferentiated tumors. It is crucial to identify potential targets to detect the features of tumor stemness and to decrease the tumor grade by targeting these markers. miRNAs, with their ability to regulate various signaling pathways, are a valuable candidate for this purpose. In this study, we explored a potential miRNA cluster, miRNAs 7/17/155, that may play role in promoting the development of high-grade breast tumors in mammary glands through EGFR-mediated EMT. Furthermore, our scRNA-seq analysis confirms that the stem cell-like cellular population in breast cancer expresses significantly higher levels of EGFR and EGFR-regulated EMT markers compared to other breast tumor clusters. Our integrative analysis reveals a compelling model wherein the miRNAs cluster 7/17/155 functions as a potential driver of high-grade, stem-like breast tumors by potentiating EGFR-mediated epithelial-mesenchymal transition (EMT).

Butner et al. (2022) demonstrated that tumor cell dedifferentiation mediates breast cancer stem cell maintenance (Butner et al., 2022). They recognized that the dedifferentiation process generates multipotent cell lineages in breast carcinomas. This multipotent stem cell population of mammary carcinoma is a critical factor in disease progression and response to therapy (Butner et al., 2022). It has been observed that the population of tumor undifferentiated stem phenotypes is significantly higher in high-grade mammary gland tumors (Tisza et al., 2016). Considering the stem nature of high-grade tumors, it would be justified not to obtain the adult stages of the MGD. Our initial in-silico analyses established a fundamental principle: the molecular footprint of MGD is progressively erased as tumors dedifferentiate. Well- and moderately differentiated (GI/GII) tumors retained significant activity of pubertal and pregnancy/lactation gene programs, including those for epithelial development and differentiation. In stark contrast, these programs were largely absent in GIII tumors, which instead exhibited a surge in inflammatory and immune-related pathways. This suggests that high-grade tumors do not merely lose differentiation; they actively suppress the transcriptional identity of the mature gland, creating a permissive environment for stemness. It seems in low-grade tumors, residual mammary gland developmental programs remain detectable due to partial preservation of differentiation, while in high-grade tumors, extensive dedifferentiation obscures these normal developmental signals.

The EMT is a complex biological process that is promoted by a series of cascades in breast tumor cells, which ultimately target the integrity of the extracellular matrix components of the tumor and lead to a change in the character of well-differentiated malignant breast tumor cells (Tisza et al., 2016; Wendt et al., 2010; Pasquier et al., 2015). EGF/EGFR signaling is a principal trigger of EMT in most solid malignancies. While other cascades, such as TGF-β and WNT, also contribute to EMT (Foroni et al., 2012), their roles are less central to the scope of our investigation. It has been detected that stimulation of EGFR leads to activation of the signal transducer and STAT3 in breast cancer cells. STAT3, through transcription factors such as slug (SNAI), affects E-cadherin expression and induces the EMT (Wang et al., 2015). On the other hand, pro-inflammatory cytokines mediated cascades, including interferon-gamma (IFN-γ), interleukin-6 (IL-6), IL-8, and tumor necrosis factor-alpha (TNF-α), are involved in tumor EMT (Suarez-Carmona et al., 2017). These cytokines, *via* activation of NF-kB signaling, influence this transformation in MG carcinoma cells (Suarez-Carmona et al., 2017). All the above mechanisms were observed in the breast tumors’ EMT process. Therefore, one of the fundamental points is which mechanisms regulate EMT in each grade of mammary tumors. Our KEGG enrichment analysis of the grade-specific DEGs did not find any significant active EMT mediators in the GI and GII tumors. In contrast, in GIII tumors, the inflammatory mechanisms and NF-kB were the active signaling pathways in the undifferentiated breast tumors. However, TGF-beta and Notch signaling pathways appeared to be deactivated (Figure 1). This finding may suggest that immune system activity, both innate and adaptive, is the leading GIII tumor-specific EMT promoter. It shows that targeting the inflammatory response could be an efficient approach to inhibit EMT and improve therapeutic responses in cases with high-grade breast carcinomas.

Due to their wild-type regulatory potential, miRNAs hold a special role in the diagnosis and treatment of malignancies. Therefore, exploring novel miRNAs or miRNA clusters that are active in tumor biological functions may pave the development of new medications. To date, specific miRNAs for undifferentiated breast tumors have been detected (Humphries et al., 2021). Plummer et al. discovered that miR-10b and miR-296b are overexpressed in high-grade breast cancers and play a significant role in the undifferentiated mammary tumor angiogenesis by stimulating VEGF expression. Blocking the activity of miR-10b and miR-296b in experimental models resulted in significant inhibition of tumor growth (Plummer et al., 2013). Their results suggest that miR-10b and miR-296b could be a fortunate miRNA cluster for targeted therapy of high-grade and undifferentiated breast tumors. Li et al. (2014) also found that downregulation of miR-140 in well-differentiated breast cancer is a critical mechanism for breast cancer stem cell development. Their molecular observation revealed that inhibiting miR-140 promotes carcinoma cell dedifferentiation by activating sRY-box transcription factor 9 (SOX9) and aldehyde dehydrogenase 1 (ALDH1) activity (Li et al., 2014). Loss-of-function of miR-145, miR-200a, and miR-205 has also been demonstrated in undifferentiated breast cancer in other studies (Götte et al., 2010; Stankevicins et al., 2017; Tsai et al., 2018). Utilizing the MGD mechanism to detect grade-specific miRNA-mRNA interaction networks in breast cancer, our findings suggest that 10 miRNAs exhibiting a gain-of-function signature may serve as potential candidates for identifying a “miRNA cluster associated with breast tumor stemness”. Enrichment analysis with KEGG pathway elevating these 10 miRNAs revealed significant affinity between this miRNA group and EMT mediators, including TGF-β signaling, ErbB (as EGFR) signaling pathway, cell adhesion molecules (CAMs), signaling pathways regulating pluripotent stem cells, and EGRF tyrosine kinase inhibitor resistance (Figure 3). Our qRT-PCR results show that the expression trend of the three miRNAs 7/17/155 increases with tumor histological upgrading. This harmonic expression pattern of miR-7, miR-17, and miR-155 indicates that their simultaneous activity may promote the dedifferentiation of breast carcinoma cells *via* a developmental pattern. Our further observations suggest that the EGFR (ERBB2) signaling pathway represents a potential central mechanism regulated by this miRNA cluster in high-grade carcinomas of the breast (Figure 4). Survival analysis further showed that high expression of miR-7 and miR-17 significantly correlates with poor outcomes (Figure 4), possibly reflecting higher metastatic potential of these breast cancers, though further studies are needed to clarify the underlying mechanisms.

MiR-155 is an oncogenic miRNA (oncomiR) detected in breast cancer (Jiang et al., 2010). Previous studies have shown its crucial role in immune cell development (Wang et al., 2021b) and regulation of inflammation in various diseases (Dickey et al., 2016; Mahesh and Biswas, 2019). Overexpression of miR-155 has been reported in tumors of MGs. In 2010, Jiang et al. found that miR-155 develops carcinogenesis in the MG by inhibiting the tumor suppressor gene suppressor of cytokine signaling 1 (socs1) (Jiang et al., 2010). They also reported that miR-155 induces its oncogenic function by activating the Janus-activated kinase (JAK)/STAT3 pathway and stimulating proinflammatory cytokines IFN-γ and IL-6 in breast carcinoma (Jiang et al., 2010). Additionally, miR-155 controls breast cancer cells’ function through the “PIK3R1-FOXO3a-cMYC axis” (Kim et al., 2018) and downregulation of cell adhesion molecule 1 (CADM1) (Zhang et al., 2019). Its cascades are even involved in the dedifferentiation of malignant cells for MGs. Zuo et al. (2018) also found that miR-155 deactivation reduced the level of breast cancer G2^+^/CD44^+^/CD90^+^ stem cells in the DA-MB-231 miR155^−/−^ cell line (Zuo et al., 2018).

Along with miR-155, miR-17 is also considered an oncomiR in breast tumors (Hossain et al., 2006). The cell cycle regulatory role of this miRNA in normal cells was reported previously. Cloonan et al. showed that miR-17–5p controls transition into the G1/S phase of the cell cycle by regulating MAPK expression (Cloonan et al., 2008). This miRNA mainly induces migration and invasion in breast cancer cells via suppressing HMG box-containing protein 1 (HBP1) gene (Li et al., 2011). The HBP1 is a DNA-binding transcription factor leading to the development of amorphous malignant phenotypes (Yu et al., 2019). According to the “results from the Norwegian Women and Cancer (NOWAC) study,” the expression level of the miR-17-5p was significantly higher in the undifferentiated breast tumors. However, this project discovered that the level of miR-17-5p was significantly decreased in luminal A tumors (Moi et al., 2019). Their findings may highlight the possible key role of miR-17 in breast cancer cell dedifferentiation and carcinoma development in the MGs.

MiR-7 is another detected oncomiR with different biological performance than miR-17 and miR-155. It suppresses cancer cell proliferation of breast and viability by activating proteasome activator subunit 3 (REGγ or PSME3) (Shi et al., 2015). The PSME3 is critical for breast cancer cell dedifferentiation and induction of EMT (Yi et al., 2017). It has been observed that overexpression of the PSME3 in malignant mammary gland cells MDA-MB -231 leads to an increase in the rate of EMT and the development of cancer stem cells (Yi et al., 2017). Although miR-7 can promote the formation of undifferentiated cells in breast tumors, Li et al. (2020) discovered that miR-7 reduced the metastatic potential of breast cancer stem cells (Li et al., 2020).

These studies highlight the need for a comprehensive study to identify miRNA clusters involved in breast tumors upgrading from a developmental biology perspective. Using the MGD mechanism consistent with the above findings, miRNAs 7/17/155 may be a potential stem breast tumor miRNA cluster. Our in-slice and qRT-PCR results showed that the activity level of all three miRNAs significantly increased in undifferentiated breast tumors compared to well-differentiated and normal tissue groups (Figure 7). We believe that the interaction of these three miRNAs through processing a series of MGD mechanisms likely initiates the formation of undifferentiated stem phenotypes in MG tumors.

The PPI analysis of mRNA intersection shows that miRNA cluster 7/17/155 regulates important networks related to EGFR/NF1/SMAD1/BACE1 (Figure 4). The prominent role of EGFR in breast cancer cells’ EMT has been demonstrated by several observations (Wendt et al., 2010; Pasquier et al., 2015). Our GSE analysis indicates that EGFR-mediated EMT is significantly more active in undifferentiated breast cancer than in well-differentiated tumors (Figure 5). Enrichment analysis of EGFR-interacted mRNAs in the GIII tumor EGFR-mediated EMT indicates to us that those genes mostly regulate biological mechanisms related to cellular adhesion and communication (Figure 5E). Our MTA assay on tissue samples also reveals that the histological level of EGFR (ERBB2) is remarkably higher in GIII tumors than in GI and normal tissue (Figure 6). Based on GSEA and MTA assessments, it may be possible to confirm that EGFR-mediated EMT is a central origin for breast tumor upgrading and generating stem population in undifferentiated tumors. Although this study requires functional validation, it is highly plausible that the miR-7/17/155 cluster regulates EGFR-mediated EMT as a regulator of stemness in high-grade breast cancer.

Detecting undifferentiated and stem cell-like populations within the breast cancer cells is crucial, as these cells are believed to play a role in tumor generation, progression, and resistance to therapy. Understanding their characteristics, biology, and behavior may lead to a more effective therapeutic approach for breast tumors. While some receptors, mainly ER, are considered the main breast cancer cell markers (Clarke et al., 2005; Buschhaus et al., 2022), the ERBB2 (HER2) receptor has also been presented as a marker for detecting high-grade cellular populations in breast carcinoma (Korkaya and Wicha, 2013). In addition, ΔNp63/p40 has been detected in a small population of tumor cells in ER^+^/HER2^+^ human breast cancers, and its presence is associated with improved patient survival. These cells exhibit a mesenchymal stem-like phenotype and are located at the tumor/stroma interface, suggesting a role for these cells in breast cancer induction or maintenance (Liu et al., 2020). We investigated EGFR-mediated EMT active markers in the high-grade breast cancer cluster (cluster 3). We observed a significant activation of this mechanism in the undifferentiated cellular cluster using scRNAseq (as shown in Figure 7). Our correlation analysis showed that the miR-7/17/155 cluster is linked to the main DEGs of the stem-like population, which are active in cellular transitions, suggesting that this cluster may be predominantly activated alongside EGFR-mediated EMT (Figure 7). However, validation through scRNA-seq multimodal approaches is still required. We also observed that the high-grade breast tumor population consists of nine distinct cell types whose trajectory genes are primarily involved in EMT regulation. This population appears to act as a central source of breast tumor progression and a niche of tumor heterogeneity, with EGFR (ERBB1) expression being markedly elevated. Taken together, these findings suggest that the miR-7/17/155 cluster, in concert with EGFR signaling, may mediate the emergence of stemness and heterogeneity in breast cancer.

## 5 Conclusion

We discovered specific miRNA-mRNA interaction developmental networks that are active in each grade of breast cancer. Our experiment identified a potential novel miRNA cluster (miR-7, miR-17, and miR-155) that may be a factor responsible for breast tumor stemness and progression. This miRNA cluster possibly induces tumor upgrading and breast carcinoma transformation through the EGFR-mediated EMT mechanism. Additionally, we observed that the undifferentiated cellular population of breast cancer, via EGFR-dependent cascades, could regulate carcinogenesis and tumor progression.

## 6 Limitations of the study

While our study provides a strong associative and bioinformatic argument, it has limitations that warrant mention. The most significant is the lack of functional validation. The definitive proof for our model would require demonstrating that simultaneous inhibition of the miR-7/17/155 cluster in high-grade breast cancer cells or patient-derived xenografts reverses the stem-like phenotype, suppresses EGFR-EMT signaling, and reduces tumorigenicity. Furthermore, the scRNA-seq analysis, while suggestive, would be strengthened by multimodal assays that directly quantify the miRNAs and their target proteins within the same single cells to cement the direct regulatory relationships.

## Data Availability

Publicly available datasets were analyzed in this study. This data can be found here: Gene Expression Omnibus (https://www.ncbi.nlm.nih.gov/geo/query/acc.cgi?acc&equals;GSE29044) ArrayExpress (https://www.ebi.ac.uk/biostudies/ArrayExpress/studies/E-GEOD-45666?query&equals;GSE45666).
