# Supplementary material for: MicroRNA cluster 7/17/155 as a potential driver for high-grade breast tumors transformation through EGFR-associated EMT
Source: Front Cell Dev Biol. 2026 Aug 14;14:1899163. doi: 10.3389/fcell.2026.1899163 (PMC13522128; doi:10.3389/fcell.2026.1899163)
Supplement: Supplementary file 1 [file DataSheet1.docx]

Supplementary Material

# 1 Supplementary Figures and Tables

## 1.1 Supplementary Table 1. Percentage of clinicopathological characteristics of qPCR cases. Patients were primarily over 45 years (64.7%), with the majority presenting at stage II (41.2%) or III (47.1%). Most tumors were ER-positive (70.6%) and over half were HER2-positive (58.8%). ER: Estrogen receptor; PR: Progesterone receptor; HER2: Human epidermal growth factor receptor 2.

| **Age (year)** | Scale | Percentage (n=27) |
| --- | --- | --- |
|  | <20: | 0.0% |
|  | 20-35: | 5.9% |
|  | 36-45: | 29.4% |
|  | 46-55: | 29.9% |
|  | >55: | 35.3% |
| **Stage (TNM)** |  |  |
|  | I | 0.0% |
|  | II | 41.2% |
|  | III | 47.1% |
|  | IV | 11.8% |
| **Histological grade** |  |  |
|  | I | 22.2% |
|  | II | 40.7% |
|  | III | 37.1% |
| **ER** |  |  |
|  | Positive | 70.6% |
|  | Negative | 29.4% |
| **PR** |  |  |
|  | Positive | 47.1% |
|  | Negative | 52.9% |
| **HER2** |  |  |
|  | Positive | 58.8% |
|  | Negative | 41.2% |
| **Triple negative** | ER^-^/PR^-^/HER2^-^ | 5.9% |

## 1.2 Supplementary Table 2. Forward primer sequences for human miRNAs. Universal Reverse Primers were obtained from Bonyakhteh Company (Bonyakhteh, Tehran, Iran). All primer specificities were verified using NCBI Primer-BLAST “<https://www.ncbi.nlm.nih.gov/tools/primer-blast/>” and miRBase “<https://www.mirbase.org/rnacentral_search/>”

| **MiRNA** | **Forward primer (5'→3')** |
| --- | --- |
| hsa-miR-7-5p | TGGAAGACTAGTGATTTTGTTGTT |
| hsa-miR-17-5p | CAAAGTGCTTACAGTGCAGGTAG |
| hsa-miR-19b-1-5p | AGTTTTGCAGGTTTGCATCCAGC |
| hsa-miR-26a-5p | TTCAAGTAATCCAGGATAGGCT |
| hsa-miR-106b-5p | TAAAGTGCTGACAGTGCAGAT |
| hsa-miR-155-5p | TTAATGCTAATCGTGATAGGGGT |
| hsa-miR-16-5p | TAGCAGCACGTAAATATTGGCG |

**1.3. Supplementary Table 3.** Clinicopathological characteristics of tissue microarray cases. **ER:** Estrogen receptor; **PR:** Progesterone receptor; **HER2:** Human epidermal growth factor receptor **IDC:** Invasive ductal carcinoma.

| **#** | **Grade** | **Stage** | **Type** | **Tumor size (cm)** | **ER** | **PR** | **HER2** | **P53** |
| --- | --- | --- | --- | --- | --- | --- | --- | --- |
| **1** | I | II | IDC- papillary | 1.8 | + | + | 1+ | 2+ |
| **2** | I | III | IDC- NOS | 1.5 | + | + | - | - |
| **3** | I | IV | IDC- NOS | 5 | + | - | 2+ | - |
| **4** | I | III | IDC- NOS | 2 | + | + | - | - |
| **5** | I | I | IDC- NOS | 1.2 | + | + | 1+ | 1+ |
| **6** | I | III | IDC- NOS | 7 | + | - | 1+ | 1+ |
| **7** | III | III | IDC- papillary | 3 | + | + | 2+ | 1+ |
| **8** | III | II | IDC- NOS | 4 | - | - | 2+ | 3+ |
| **9** | III | II | IDC- NOS | 4 | - | - | - | - |
| **10** | III | III | IDC- NOS | 4 | - | - | 1+ | - |
| **11** | III | II | IDC- medullary | 3.5 | - | - | - | - |

1.4	**Supplementary Figure S1**. scRNA-seq normalization, differential gene expression, and clustering visualization in the PCA. A) Quality control metrics for genes, reads, and mitochondrial transcripts per cell as determined by standard Seurat pre-processing. Violin plots illustrate the nFeature_RNA, the nCount_RNA, and the percent.mt. B) Scatter plot showing the relationship between nCount_RNA and nFeature_RNA after quality control. C) PCA displaying the distribution of filtered cells according to their mitotic phase status. D) Cell-to-cell variation of each feature, with each red point representing a highly variable feature. Black dots indicate non-variable features, while red dots indicate variable features. E) Heatmaps of the top 15 principal components explaining the primary sources of heterogeneity in the retinal scRNA-seq data. F) Cluster-based visualization of scRNA-seq data using PCA.
